# Health Assessment and the Right to Health in Sweden: Asylum Seekers’ Perspectives

**DOI:** 10.1371/journal.pone.0161842

**Published:** 2016-09-02

**Authors:** Lubin Lobo Pacheco, Robert Jonzon, Anna-Karin Hurtig

**Affiliations:** 1 Dept. of Public Health and Clinical Medicine, Umeå University, 901 85 Umeå, Sweden; 2 The Public Health Agency of Sweden, Solna, Sweden; University of North Carolina at Chapel Hill, UNITED STATES

## Abstract

**Background:**

Swedish law entitles asylum seekers to a voluntary health assessment and to “health care that cannot be postponed”. The last expression suggests, however, restrictions on the entitlement, and what it may or may not include remains ultimately a decision for health professionals in the specific case. Indeed, the health assessment constitutes the sole active effort from Swedish authorities to fulfill this right. This study was therefore aimed at assessing how the information, procedures and services related to the health assessment are accessible and acceptable to fulfill the right to health of asylum seekers, from their own perspective.

**Methods:**

The study has a cross-sectional design. A questionnaire was administrated in 16 language schools for immigrants, in four counties of Sweden. Three hundred eighty-six individuals fulfilled the inclusion criteria. The frequency of their answers was tabulated to estimate how the information, procedures and services related to the health assessment correspond to the criteria for accessibility and acceptability regarding the right to health.

**Findings:**

Forty-eight (12.4%) respondents did not undergo the health assessment. Thirty-one of them did not even receive the invitation letter. They said they lost the opportunity to know their health status, to obtain treatment for or advice about their health problems. Additionally, 55.2% of those who attended the health assessment indicated that their needs were overlooked, particularly when these were of a psychological nature. Two in three participants also considered the health assessment to be a communicable disease control, rather than an effort to take care of their health needs. Nevertheless, the respondents had a positive attitude towards the health assessment as such.

**Conclusions:**

Although being an important contribution, the health assessment does not suffice to fulfill the right to health of asylum seekers because there are shortcomings regarding the accessibility and acceptability of the information, procedures and services that it includes.

## Introduction

Natural disasters, war and violence force many people to leave their home and seek protection in other countries. UNHCR reported that by mid-2015 over 20 million people had crossed international borders for such reasons, and that 678,734 of them had reached EU countries as asylum seekers [1]. Similarly, figures from the Swedish Migration Agency show that the number of asylum applications issued in the country increased sharply to reach 162,877 at the end of the same year [2]. Forced migration implies difficult journeys accompanied by various health threats. This is particularly noticeable regarding asylum seekers since the uncertainty surrounding the asylum process affects even more their already vulnerable health [3–5].

The health of refugees and asylum seekers is a human rights concern protected by international law. For example, the International Covenant on Social, Economic and Cultural rights (article 12) declares “the right of everyone to the enjoyment of the highest attainable standard of physical and mental health” [6], and the 1951 Refugee Convention emphasises that refugees should enjoy access to health services equivalent to that of the host population [7]. Likewise, the Convention on the Elimination of all forms of Racial Discrimination insists on States’ obligations to “ensure […] the right of non-citizens to an adequate standard of physical and mental health by, inter alia, refraining from denying or limiting their access to preventive, curative and palliative health services”[8]. At the regional level, the EU Minimum standards on the reception of applicants for asylum in Member States, updated in 2009, state in Art. 19 that asylum seekers must receive “the necessary health care which shall include, at least, emergency care and essential treatment of illness or mental disorders”. It also says that those with special needs must receive medical or other assistance, including appropriate mental health care when required [9].

International human right treaties create obligations on signatory States since its dispositions are expected to become part of the domestic legislation. Accordingly, the right to health of asylum seekers is ruled in Sweden by the Health and Medical Care for Asylum Seekers and Others Act [10]. This law entitles asylum seekers to dental and health care, maternity and childbirth care, contraceptive advice and abortion. The scope of the entitlement is referred to as “health care that cannot be postponed”. The meaning of this expression is, however, imprecise. Some understand it as acute or emergency care only, while others attribute a broader meaning to it. They argue that health problems, whether acute or not, may become severe, chronic or fatal if they are not treated. In an attempt to clarify the concept, the Swedish Board of Health and Welfare concluded that it is unethical, medically impossible or inappropriate to list certain diagnoses, conditions, or operations as covered therein [11]. In any case, the term suggests restrictions on the entitlement, and what it may or may not include remains ultimately a decision for health professionals in the specific situation [12]. The law also makes county councils accountable for inviting the asylum seeker to a voluntary health assessment, which in fact becomes the sole active effort from health services to reach out to all asylum applicants and deal with their health concerns.

### Health Assessment of Asylum Seekers

The Swedish Board of Health and Welfare indicates that the health assessment has a double purpose: on the one hand, to identify health problems demanding immediate attention in the best interest of the individual, and on the other, to identify and take appropriate measures to prevent the spread of contagious diseases, as a public health measure [13]. However, scholars have pointed out an imbalance in practice between these two purposes [14,15].

The health assessment of asylum seekers has been consolidated over the years in relation to several factors, among them, the spread of contagious diseases, such as HIV (human immunodeficiency virus), STI (sexual transmitted infections) and TB (tuberculosis) [16], the countries of origin and the number of asylum seekers, as well as the diversification of accommodation alternatives. Since the 1980s, Swedish authorities have stressed the importance of HIV test within the health reception programs [17]. In the early 90s, when most applicants were housed in state-operated refugee camps, full-time nurses performed the assessments in situ. They also acted as the first contact in the health care chain and decided on further measures to meet the health needs of those living in the camps [18]. But other accommodation forms such as private housing increased after 1994, making it possible for asylum applicants to lodge with relatives who had moved to Sweden earlier [19]. These new conditions required continuous adjustment of the system, and currently, the health assessments are carried out mainly in primary health care centres.

Since 2008, asylum seekers must, by law, be offered a health assessment upon arrival. Migration officials are the first ones to inform the applicants about this right. Subsequently, health providers are responsible for inviting to, carrying out the assessment, and providing complementary remedies, in line with the concept “health care that cannot be postponed”. Even so, the health assessment is voluntary for the individual, who can decide whether to accept the offer or not. Some studies have shown that asylum seekers and other immigrants tend to avoid contact with the health services, fearing to be tested for HIV in the consultation. These studies indicate that they are afraid of being stigmatized, socially isolated [20] or even deported [21] as a consequence of a HIV-positive diagnosis.

The health assessment is the first encounter with the Swedish health care system for most asylum applicants, and probably the only one for those whose application is not approved. Nevertheless, the Swedish Association of Local Authorities and Regions has repeatedly reported that scarcely 50 percent of asylum seekers undergo this assessment [22–24]. The reasons behind the low rate are not yet clear, though explanations related to structural and organisational shortcomings have been suggested [22,25]. In any case, few attempts have been made to explore this phenomenon from the users’ perspective. Studies by Lindkvist et al. [20] and Nkulu et al. [21], for instance, refer to immigrants’ encounters with health services but not specifically to asylum seekers. Little is known about how they experience the health assessment, or the extent to which they consider that it contributes to accomplish the fulfillment of their right to health. This study therefore aims to assess the health assessment from the asylum seekers’ perspective.

### Conceptual Framework

The right to health implies liberties and entitlements. The liberties include the right to control one’s health and body, and sexual and reproductive freedom, and the right to be free from interference, such as torture, non-consensual medical treatment and experimentation. The entitlements include the right to a health system that provides equal opportunities for everyone to enjoy the highest attainable level of health [26].

To monitor its implementation, the rights have to be broken down into operational indicators [27]. Thus, the Committee on Economic, Social and Cultural Rights (CESCR) proposed four interrelated dimensions to analyse the right to health. These are *availability*, *accessibility*, *acceptability and quality* [26]. The analysis of all four of these dimensions is beyond what is feasible for this study. Therefore, we decided to focus exclusively on *accessibility* and *acceptability* because these aspects are particularly relevant when users and providers of health services meet across national and cultural borders, such as when asylum seekers have their health assessments.

1. Accessibility means the possibility for everyone to reach health facilities, goods and services without discrimination. This dimension has four overlapping aspects:
  - Accessibility without discrimination. Health facilities must, in law and in fact, be accessible to all, especially the most vulnerable groups, without discrimination because of race, sex, language, religion, political or other opinion, national or social origin, property, birth, physical or mental disability, health status, sexual orientation and civil, political, social or other status (CESCR, 2000, paragraphs 12 and 18).
  - Physical accessibility. Health facilities, goods and services must be physically reachable by the population, especially the vulnerable groups.
  - Economically accessible. Governments must ensure that health facilities, goods and services, whether privately or publicly provided, are affordable for all, including socially disadvantaged groups.
  - Accessible health-related information. This comprises the right to seek, receive and divulge information about health, and the right to have personal health data treated with confidentiality.
2. Acceptability implies two aspects: respect for medical ethics and cultural appropriateness. Health services should be designed to respect confidentiality and improve the health of patients, but they should also be culturally appropriate. This refers to health providers’ respectfulness, level of cultural understanding and acceptance of the patient and his/her way of life. It also means sensitivity to gender and age requirements.

## Method

### Study-design and Settings

The study has a cross-sectional design. Data was collected in language schools for former asylum seekers and other migrants during the spring of 2013, in four counties of Sweden (Norrbotten, Skåne, Östergötland and Stockholm). There were 132 language schools in these counties in 2011, according to the Swedish National Agency for Education. Out of these, 20 were randomly selected and invited to participate in the study. Finally, 16 schools agreed to take part.

### Study Participants

Eligible participants for this study were language students who had been asylum seekers. According to Swedish law, all asylum seekers should be offered a health assessment. We therefore assumed that all former asylum seekers at the language school had received such an offering. Focusing on this particular group was a deliberate decision based on both ethical and methodological reasons. We thought that questioning current asylum seekers on their experience of the health assessment could increase the anxiety produced by the pending decision on asylum [3,4], which, in turn, could affect the consistency of their answers. In addition, it was likely that some of them had not yet undergone the health assessment, and therefore, would not be able to answer the questionnaire. We also considered, initially, including individuals whose asylum applications had been rejected but this was later discarded because of difficulties in reaching them, and because the anxiety provoked could be higher due to their particular situation [28,29].

A second inclusion criterion was that the participants had sought asylum in 2010 or later. The reason for excluding earlier applicants was that the law on health care for asylum seekers was sanctioned in 2008. Prior to this law, there was no obligation to offer the health assessment, and the counties were not equally committed to it [22]. The 2008 law was gradually implemented and, we assumed that it was in force in all counties by 2010.

However, information about the students’ reasons for migrating and year of arrival was not possible to be obtained prior to the survey, since the schools did not register this data. Moreover, the disclosure of reasons for migration is considered to be a sensitive matter, particularly concerning refugees and asylum seekers. To overcome these difficulties, the questionnaire included some questions that allowed us to identify and separate the group of interest for the study, namely, asylum seekers who arrived after 2009.

The questionnaire was translated into the languages spoken by the largest groups of asylum seekers in Sweden in 2010 and later, namely, Arabic, Somali, Tigrinya and Farsi. It was also translated into the most prevalent European languages (English, French and Spanish) based on the consideration of the expansion of these languages worldwide and that some asylum seekers might have come from countries where these languages are spoken.

**Fig 1:**
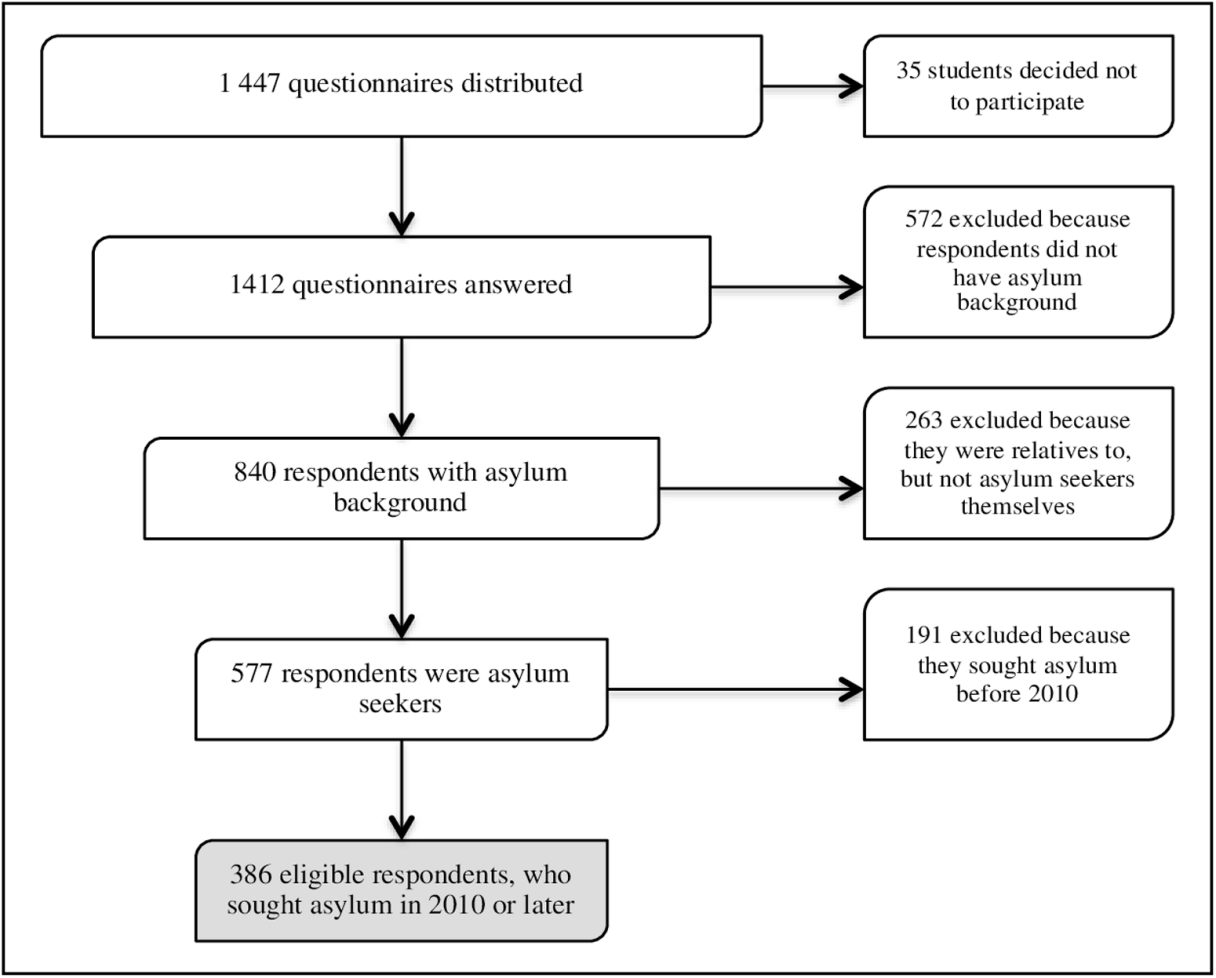
Flow diagram of respondents of the survey and eligible for the study.

In total, 1,447 questionnaires were distributed within 95 class groups. After receiving the initial information, 35 students decided not to participate, while 1,412 (97.6%) answered the questionnaire. Out of them, 890 were in Arabic, Somali, Farsi and Tigrinya, and 522 were in the other languages. Five hundred seventy-seven of the respondents came as asylum seekers, and 263 came as relatives to asylum seekers. The rest, 572 respondents, came to Sweden for other reasons, such as work, studies or establishing a relationship with a Swedish citizen. Among the 577 former asylum seekers, 191 came to Sweden before 2010, and 386 arrived in 2010 or later. Thus, these 386 individuals, who were within an age range of 18–65 years, fulfilled the inclusion criteria for this study, namely, being a language student, having sought asylum in Sweden in 2010 or later, and mastering at least one of the languages used in the questionnaire (Fig 1).

### Data Collection Instrument and Procedure

The questionnaire consisted of 51 items. Some had been used in the Swedish national public health survey “Health on equal terms"; others were elaborated by the researchers based on interviews with health care providers, discussions with target groups and relevant literature about health assessments [30–32] and the right to health [33]. Most of the items were closed-ended questions, related to six topics: (1) socio-demographic background, (2) general knowledge and perceptions about health and health care, (3) information received prior to the health assessment, (4) knowledge and perceptions about the health assessment’s purpose and contents, (5) communication with health care providers and others involved in the health assessment and (6) the health assessment in relation to individual health needs. Questions about the reasons for migration and the year of entry to Sweden were also included in the questionnaire. These allowed the respondents with an asylum background to be diverted to the specific questions on the health assessment and their answers to be sorted out during the data analysis.

The questionnaire was initially produced in Swedish and was translated into the other languages. The translated versions were piloted with migrant students at language schools in Stockholm and were adjusted, according to their suggestions, before being used. Language supporters were available for those students who could not read and write the respective language, but wanted to participate. These supporters were persons who mastered the language of the respondent and had either been working as interpreters or had experience in research.

### Analysis Strategy

The study focuses on the health assessment because it constitutes the sole intended and systematic effort by Swedish authorities to deal with the health of asylum seekers. We analyse the extent to which the respondents considered the information, procedures and services related to the health assessment to be accessible and acceptable, because these are essential dimensions of the right to health, according to the CESC. *Accessibility* was analysed by three variables: universal access, language and communication, and health-related information. *Acceptability* was analysed by the variable: cultural appropriateness, it means respect for cultural differences. Frequency distributions were calculated using the statistical package Stata 13.0.

### Ethical Considerations

Ethical clearance for the study was obtained from the Regional Ethical Review Board in Umeå (Registration number 2014-11-32M). Permission was also acquired from the principal of each school. The respondents were informed, verbally and in written, about the purpose of the study. They were also told that participation was voluntary and that they could withdraw from the study at any time without explanation. Likewise, it was explained that the answers were to be given anonymously and that no individual could be identified in the results or reports.

## Results

### Socio-demographic Characteristics

The study is based on 386 individuals who sought asylum in 2010 or later. Their ages varied between 18 and 65 years (mean 32), and 167 (43.3%) were females. Regarding their origin, 129 (33.4%) of the respondents came from Somalia, 86 (22.3%) from Syria, 49 (12.7%) from Eritrea, 45 (11.7%) from Afghanistan, 24 (6.2%) from Iraq and 53 (13.7%) from other countries. At the time of the survey, 308 respondents (79.8%) had been in Sweden up to two years. More precisely, 207 (53.6%) reported that they had arrived within the last 12 months. Likewise, 222 of the participants (57.5%) indicated that they had done the health assessment during the last year. Detailed information about the study population is presented in Table 1.

**Table 1:** Respondents’ socio-demographic characteristics by country of origin (N = 386).

	N = 45	N = 49	N = 24	N = 129	N = 86	N = 53	N = 386
**Sex**							
Female	20 (44.4)	18 (36.7)	13 (54.2)	72 (55.8)	26 (30.2)	18 (33.9)	167 (43.3)
Male	25 (55.6)	30 (61.2)	11 (45.8)	53 (41.1)	56 (65.1)	34 (64.1)	209 (54.1)
Missing (N/A)	-	1 (2.0)	-	4 (3.1)	4 (4.6)	1 (2.0)	10 (2.5)
**Age**							
18–24	20 (44.4)	7 (14.3)	9 (37.5)	26 (20.1)	20 (23.3)	10 (18.9)	92 (23.8)
25–34	13 (28.9)	17 (34.7)	7 (29.2)	56 (43.4)	31 (36.0)	23 (43.4)	147 (38.1)
35–44	7 (15.6)	17 (34.7)	7 (29.2)	18 (14.0)	19 (22.1)	14 (26.4)	82 (21.2)
45–54	4 (8.9)	5 (10.2)	1 (4.1)	16 (12.4)	6 (7.0)	2 (3.7)	34 (8.9)
55–65	1 (2.2)	1 (2.0)	-	3 (2.3)	8 (9.3)	1 (1.9)	14 (3.6)
Missing (N/A)	-	2 (4.1)	-	10 (7.8)	2 (2.3)	3 (5.7)	17 (4.4)
**Education**							
Analphabet	20 (44.4)	2 (4.1)	1 (4.2)	39 (30.2)	6 (7.0)	-	68 (17.6)
1–6 years	15 (33.3)	10 (20.4)	10 (41.6)	42 (32.6)	18 (20.9)	9 (17.0)	104 (27.0)
7–12 years	3 (6.7)	28 (57.2)	9 (37.5)	27 (20.9)	24 (27.9)	19 (35.8)	111 (28.8)
13+ years	6 (13.4)	8 (16.3)	4 (16.7)	13 (10.1)	38 (44.2)	24 (45.3)	93 (24.0)
Missing	1 (2.2)	1 (2.0)	-	8 (6.2)	-	1 (1.9)	10 (2.6)
**Marital status**							
Single	21 (46.7)	25 (51.0)	13 (54.1)	5 (3.9)	45 (52.3)	30 (56.6)	139 (36.0)
Married	18 (40.0)	19 (38.8)	10 (41.7)	70 (54.3)	36 (41.9)	22 (41.5)	175 (45.3)
Cohabitant	-	1 (2.0)	-	36 (27.9)	-	-	37 (9.6)
Divorced	2 (4.4)	3 (6.0)	-	6 (4.7)	4 (4.7)	-	15 (3.9)
Widowed	3 (6.7)	1 (2.0)	1 (4.2)	5 (3.9)	1 (1.1)	1 (1.9)	12 (3.1)
Missing	1 (2.2)	-	-	7 (5.4)	-	-	8 (2.1)
**Children**							
No children	24 (53.4)	26 (53.1)	16 (66.7)	55 (42.6)	47 (54.6)	27 (50.9)	195 (50.5)
In Sweden	17 (37.8)	10 (20.4)	7 (29.2)	37 (28.7)	27 (31.4)	17 (32.1)	115 (29.8)
In other countries	2 (4.4)	12 (24.5)	-	32 (24.8)	9 (10.5)	9 (17.0)	64 (16.6)
In Sweden and other countries	1 (2.2)	-	1 (4.1)	2 (1.6)	-	-	4 (1.0)
Missing	1 (2.2)	1 (2.0)	-	3 (2.3)	3 (3.5)	-	8 (2.1)
**Religion**							
Muslim	43 (95.6)	17 (34.7)	11 (45.9)	128 (99.2)	54 (62.8)	33 (62.3)	286 (74.1)
Christian	-	31 (63.3)	3 (12.5)	-	28 (32.6)	14 (26.4)	76 (19.7)
Atheist	2 (4.4)	1 (2.0)	-	-	4 (4.6)	4 (7.5)	11 (2.8)
Other	-	-	8 (33.3)	-	-	1 (1.9)	9 (2.3)
Missing	-	-	2 (8.3)	1 (0.8)	-	1 (1.9)	4 (1.1)
**Health assessment**							
Yes	35 (77.8)	48 (97.9)	17 (70.8)	119 (92.2)	73 (84.9)	46 (86.8)	338 (87.6)
**No**	**10 (22.2)**	**1 (2.1)**	**7 (29.2)**	**10 (7.8)**	**13 (15.1)**	**7 (13.2)**	**48 (12.4)**

### The Health Needs of Asylum Seekers

One hundred ninety-six respondents (50.8%), slightly more men than women, reported suffering health problems at the time of arrival in Sweden. They were described as psychological disturbances, reported by 32 respondents (16.3%); physical problems or injures were mentioned by 26 persons (13.3%); pregnancy by 14 (7.1%); influenza by 19 (9.7%); chronic diseases by 16 (8.2%); dental problems by 7 (3.6%); infections, 8 (4.1%); and other health needs by 23 (11.7). Additionally, 305 of the respondents (79%) indicated that they personally or someone in their family had been exposed to violence in their home countries.

One hundred eighty-eight respondents (48.7%) lived together with other asylum seekers during the asylum process. Nevertheless, 225 (58.3%) reported feelings of loneliness and isolation, and 129 (33.4%) reported having no one with whom to share their worries or problems.

### Accessibility

#### Universal access

**Fig 2:**
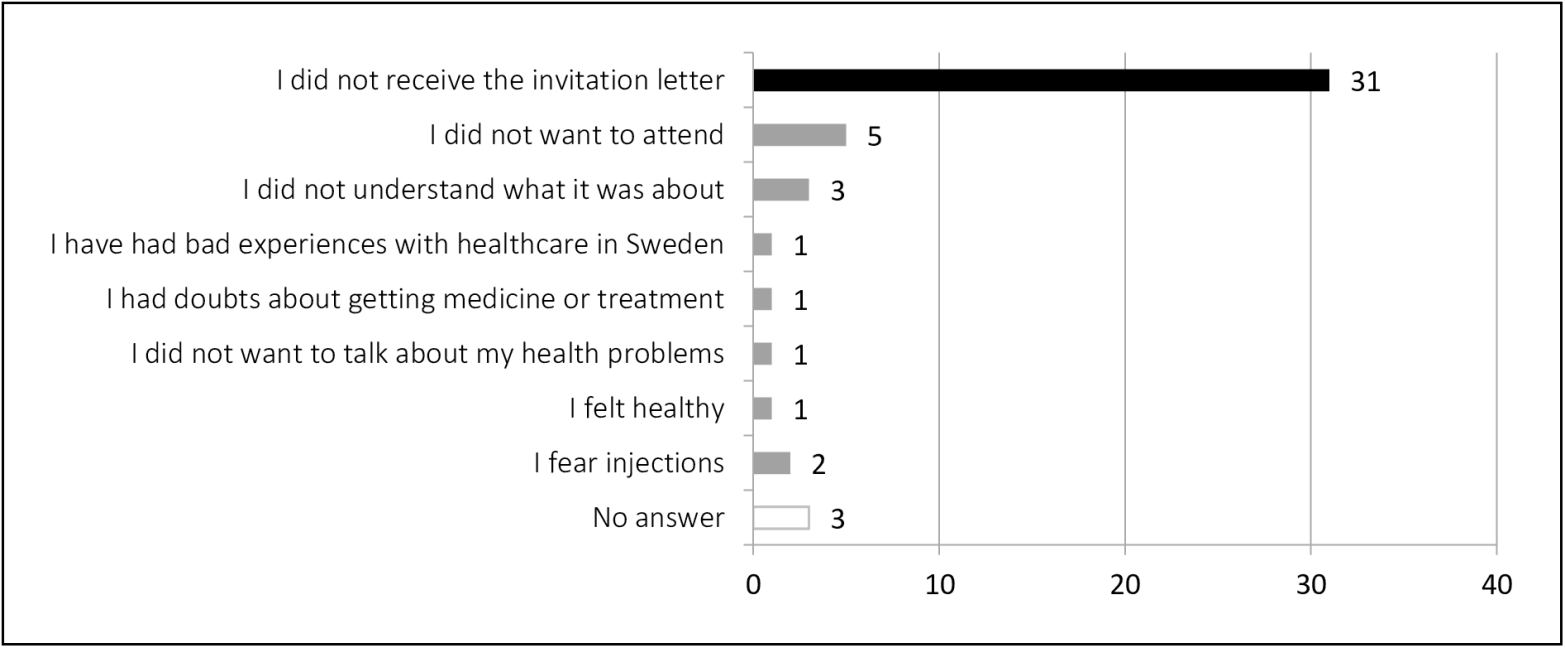
Respondents’ reason for not attending the health assessment (n = 48).

Table 1 shows that 48 respondents (12.4% of total) did not undergo the health assessment. Amongst them, 31 (64.6%) indicated not having received the letter of invitation. Among those who received the letter, five respondents (10.4%) said they simply did not want to attend, and three others (6.2%) that they did not understand what it was about. Others described previous bad experiences with health care providers in Sweden, doubts about getting medicine or treatment, feeling healthy or uncomfortable when talking about their own health problems, as well as fears of syringes (Fig 2).

In any case, the respondents who did not undergo the health assessment complained about having lost the opportunity to know their health status and to obtain treatment for or advice about their health problems.

#### Language and communication

Among the 338 respondents (87.6%) who attended the health assessment, only 302 (78.2%) reported having received the invitation letter. Of them, 293 (97%) also indicated that the letter was issued in Swedish, but just 93 (31.7%) stated that they understood the content. Similarly, 108 of those who attended the health assessment (31.9%) said they had not been informed that the assessment was a right or that they could abstain from it. Moreover, 98 individuals (29%) believed that the assessment could influence the decision on asylum, and 87 (25.7%) said that they had not been informed that they, as asylum seekers, had limited access to health services.

Regarding the communication with health care personnel, 269 respondents (79.6%) indicated that interpreters were provided. However, only 170 of them (63.2%) said having understood what the doctor or nurse talked about. Similarly, 241 respondents (71.3% of those attending the assessment) reported that samples were taken, but only 171 (50.6%) knew what kind of samples, and even fewer, 149 (44.1%), knew the results. Additionally, 100 individuals (29.6% of those attending the assessment) said they had not had the opportunity to express their own health concerns. They also complained about the short time assigned for the assessment.

#### Access to health-related information

Two hundred fifty-nine individuals (76.6%) of those who attended the health assessment considered it to be primarily a communicable disease control, rather than oriented toward other health needs. Nevertheless, almost one third of them indicated that they had not received preventive information about HIV, STI or TB, or information about contraception and family planning. Likewise, 76 respondents (29.3%) said that they did not know whom to contact if they needed psychological support.

### Acceptability

#### Unattended health needs

**Fig 3:**
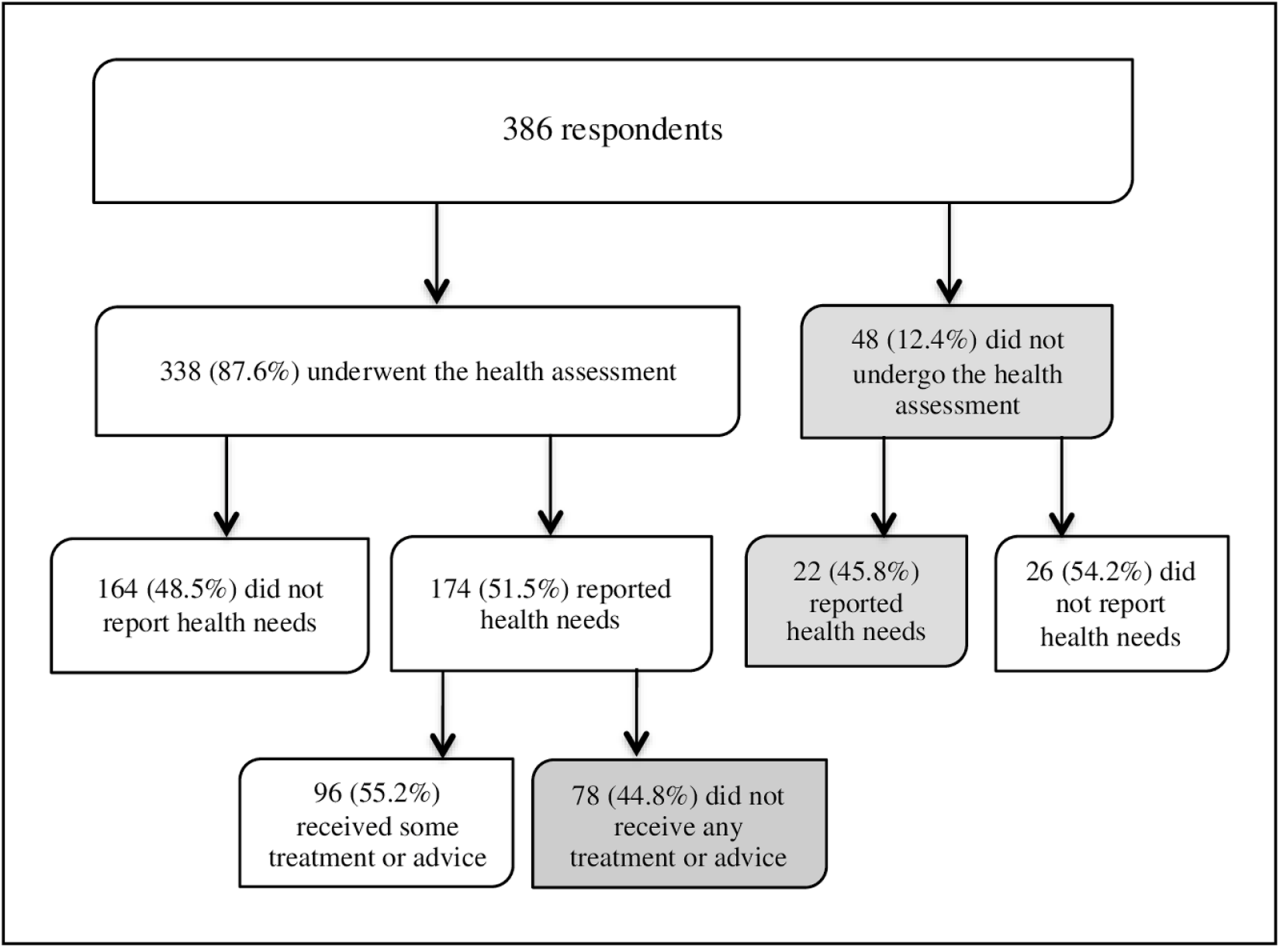
Flow diagram of health assessments and perceived health needs among the participants in the study.

One hundred ninety-six respondents (50.7% of the total) reported that they had health problems during the first months after arrival. Of them, 174 (88.8%) underwent the health assessment, and 22 did not. However, only 96 individuals (55.2%) of those who attended the assessment indicated receiving some treatment or advice (Fig 3).

Health care was often denied when it had to do with psychological disturbances, as indicated by 21 (65.6%) of the 32 respondents who reported such needs. In contrast, more attention was paid to individuals who expressed other kinds of needs. For instance, four (25%) of 16 respondents suffering from chronic diseases said they did not receive treatment, and only one (5.5%) among those affected by influenza (18 respondents), while no one in the group affected by infectious diseases (8 persons) made such complaints. Likewise, 100 respondents (29.6%) indicated that they were not given a chance to express other health-related concerns, such as headaches, pain or war-related trauma.

#### Cultural appropriateness

Although their ethnic and cultural differences with the health care providers, most of the respondents reported that they had been treated respectfully during the health assessment. However, 37 (10.9%) diverted from this opinion. They expressed distrust in the person who carried out the health assessment and indicated feeling offended or insulted. They considered being treated inappropriately due to their language difficulties, having another ethnic, religious or cultural background, or because of gender and age differences with the health care provider. Moreover, they reported that the health assessment in no manner contributed to improve their health.

## Discussion

To our knowledge, this is the first study that focuses on the health assessments and the right to health of asylum seekers, from their own perspective. Our goal was to assess the role of the health assessment in fulfilling their right to health. The results show that not all of the respondents had access to a health assessment, even though they were asylum seekers. Some were neither offered nor underwent the assessment. In addition, many respondents considered the health assessment primarily as an infectious disease control rather than an opportunity to express their health concerns. We also found that cultural differences were not properly met, giving raise to misunderstandings and feelings of discriminations, which undermines the acceptability of the health assessment among the target groups.

Our findings are consistent with previous studies indicating difficulties in the provision of health assessments to asylum seekers. Hjern and Allebeck identified, in 1997, the heterogeneity of the content of the health assessment among the different providers, when refugee camps were the most common form of accommodation [18]. Since then, the Swedish authorities have attempted to harmonise the contents and procedures of the health assessment. The 2008 law on health care for asylum seekers constitutes a clear effort in this regard. However, this issue does not seem to be equally prioritised among county councils, which are responsible for the provision of health services in Sweden [23–24].

Like Hjern and Allebeck [18], we also found that most asylum seekers are willing to participate in the health assessment. When they did not participate, it was commonly because of not having received the invitation letter, rather than them rejecting it. One explanation for the difficulties in reaching individuals with the letter could be the diverse forms of accommodation that have been developed over the years [17,19]. Those living in camps are probably easier to reach than those housed temporary in private homes with relatives or friends, or those living on their own. However, this issue was not the purpose of this study and it requires further research.

In our study, only 17 individuals (4.4% of the total) seemed to have deliberately avoided the health assessment. Some argued that they felt healthy or that they simply did not want to attend. These explanations sound reasonable, especially considering the voluntary character of the health assessment. Other studies suggest, however, that migrants tend to avoid contact with health services in Sweden because of fearing to be tested for HIV during the consultation. They also indicate that immigrants are afraid of being stigmatized, socially isolated [20] or even deported [21] as a consequence of a HIV-positive diagnosis. Nevertheless, our results primarily point to structural shortcomings, rather than to individual decisions of rejecting the offer.

The proportion of the respondents who did not undergo the health assessment (12.4%) is low in comparison to official statistics at national level [22–24]. The difference might be due to our sample size, and to the fact that our study is based on only four of the 21 Swedish counties. All the same, our results show that not all asylum seekers have access to the health assessment, although it is stated to be their right by Swedish law. Because the health assessment is the sole active effort from health services to deal with the health needs of asylum seekers, their right is violated when they are not reached by an invitation, regardless of the reason. Additionally, they are denied access to health information and the health care to which they are entitled. As some of the respondents indicated, they lost the chance to know their health status and to receive care, treatment or advice.

Our results also suggest a lack of clarity regarding the purpose of the health assessment within the target groups. Almost one-third of the participants believed that the health assessment could influence the decision on asylum. A similar proportion believed that attending the assessment was mandatory. The respondents also indicated that the content of the health assessment did not properly meet their expectations and needs. Many experienced it as a control for communicable diseases, rather than an opportunity to express their particular health concerns and to receive treatment. They complained that their health needs were overlooked when they referred to problems other than HIV, STI and TB. This suggests an imbalance between the twofold objective that the National Board of Health and Welfare has ascribed to the health assessment, i.e., to identify the individual’s health needs that require immediate attention and to detect contagious diseases from a public health interest [13].

Even when the control of communicable diseases seems to be in focus, more attention has been paid to take samples than to inform individuals about how to prevent such diseases. One-third of the participants in our study reported that they did not receive this information. Thus, the health assessment does not fulfill the purpose of providing access to preventive health-related information, which also was pointed out in a previous study [34].

The failure to adequately meet the health needs of the respondents, particularly when these were of a psychological character, is a matter of concern. Previous studies have shown that the asylum process adds new stressors to the applicants’ already vulnerable health [3–5]. Because the health assessment is usually carried out during the asylum process, it is unacceptable that psychological needs are not given sufficient attention. Some may argue that the health assessment is not meant to be a comprehensive examination, and that it cannot deal with every single complaint expressed by the individual. Others may also say that the law restricts the entitlement to “care that cannot be postponed”. We argue, however, that more attention should be paid to the individual’s needs, because the health assessment is the first encounter with the Swedish health care system, and probably the only one for those whose application is rejected. Further, we believe that remedies may be more effective and the negative consequences reduced if health problems are identified and treated at an early stage.

The concept of “care that cannot be postponed” is, in our perspective, problematic because it turns migration status into a determinant factor for the legal entitlement to health care. It challenges the basic principles of medical ethics, because doctors and nurses are expected to consider the patient’s migration status when providing health care. This does not seem to be in harmony with international treaties that sanction the right to health of the most vulnerable groups, without discrimination [6–9]. Thus, the limitations on asylums seekers’ access to health care, although legally grounded, can be morally questioned because introducing discriminatory practices that erode the basic principles of health care, e.g., care according to the needs, and principles of human rights, e.g., the equal value of all human beings [33].

Additionally, some of the respondents also indicated that they had felt disrespected, offended or discriminated against at the health assessment because of the attitude of the health personnel. This may be due to shortcomings and misinterpretations in the cross-cultural communication, when providers and users belong to different cultural contexts. Nonetheless, perceived discrimination is relevant to one’s health status. A study from USA found that patients who perceived racial discrimination while seeking health care in the past year were at greater risk for poor health [35]. Other suggests that feelings of discrimination may negatively impact the individual’s assessment of quality of care received, and that such feelings may lead to decreased adherence to medical advice and medication regimens [36]. Individuals may also consider the health services to be inadequate and, in consequence, refrain from seeking health care even when feeling seriously sick [37–39]. Regarding asylum seekers, this can lead also to discourage others from attending the health assessment.

According to the CESCR, non-discrimination is the first dimension of accessibility of the right to health [26]. Besides, culturally inappropriate interventions undermine the acceptability of the health assessment, as a service, and may erode asylum seekers’ confidence in the entire health care system. Therefore, measures should be taken to reduce the risk of both actions and omissions that can be perceived as discrimination, neglect or mistreatment [40].

In summary, failing to undergo the health assessment may represent negative consequences for the individual, as well as for society. At the individual level, not being assessed means ignoring one’s own health status and missing the opportunity to ask for and to receive health care. It also means not having access to information that is useful to maintain or improve one’s own health and to prevent illness. At a societal level, it has public health implications because of a higher risk of spreading communicable diseases. Additionally, individuals’ lack of knowledge about how to navigate within the health care system may lead to misusing services and increasing costs because they may unnecessarily demand services, or seek such services late, perhaps even too late.

### Limitations of the Study

One limitations of our study is that it focuses on individuals who already had obtained a residence permit. This may introduce selection bias since their experience may differ from that of current applicants and rejected ones. Individuals’ perception probably modifies in the aftermath of the asylum process, becoming more positive among those granted asylum than among those being rejected. It is likely that rejected applicants would have reported even more negative experiences than what we found, particularly regarding distrust in authorities. However, excluding these individuals, as well as current applicants, was a deliberate decision for methodological and ethical reasons.

There may also be recall biases because the data were gathered retrospectively, and the participants might have mixed their experience of the health assessment with other encounters with health services. To reduce this risk, we decided to only include individuals who had applied for asylum in 2010 or later. In fact, most respondents (79.8%) had been in Sweden for two years or less at the time of the survey, and many of them (57.5%) had experienced the health assessment within the last 12 months. Besides, medical consultation does not occur very often in the population at large, and it is less frequent among immigrant groups, where researchers rather have found a refraining behavior [35,36].

Notwithstanding, research addressing the health assessments and the right to health of asylum seekers, from their own perspective, is still limited. This study may therefore serve as a base for further studies.

## Conclusions and Recommendations

This study suggests that the health assessment, although being an important contribution, does not suffice to fulfill the right to health of asylum seekers. The respondents expressed positive opinions about the health assessment as such, but they also indicated structural shortcomings regarding the information, procedures and services it implies. This undermines the accessibility and acceptability of the health assessment, which are two fundamental dimensions of the right to health. The achievement of this right can be improved by several measures, among others: increasing efforts to reach all asylum seekers with the invitation letter; a better balance of the double purpose of the health assessment by considering the needs of the individual beyond the concept of “care that cannot be postponed”; increasing awareness among health care providers on the challenges of cross-cultural communication; and giving asylum applicants more clear information about the purpose and content of the health assessment, its voluntary character, and on how to navigate within the Swedish health care system.

## Supporting Information

S1 Database Right to health. (DTA)

S1 Questionnaire (PDF)
