# Supplementary material for: Health Assessment and the Right to Health in Sweden: Asylum Seekers’ Perspectives
Source: PLoS One. 2016 Sep 2;11(9):e0161842. doi: 10.1371/journal.pone.0161842 (PMC5010180; doi:10.1371/journal.pone.0161842)
Supplement: S1 Questionnaire — (PDF) [file pone.0161842.s002.pdf]

Hi!

Do you want to participate in a study?

This study is about the right to health for immigrants in Sweden. It is aimed to persons who recently have come here and probably have been invited to a free health examination. We want to know what you think about the health examination and your experience of it, if you had one done.

The objective of the study is to highlight your perceptions and experiences of the health examination and the Swedish health care system.

You can participate in the study by answering this questionnaire. Participation is voluntary, but your opinion is important in order to improve health examinations and to get more people attending the examination.

No personal information that may reveal your identity is requested. Your answers will be grouped with those of other participants, in order to secure secrecy.

If you do not understand a question, ask for support from the person in charge of the survey. If you consider a question to be sensitive and that you do not want to answer it, you may just leave it behind and go to the next question.

Finally, we want to emphasise that your participation is very important and that you contribution will make this study more valuable. If you still do not want to participate in the study; please, return the questionnaire to the person in charge.

Thanks a lot for your collaboration.

Best regards,

Lubin Lobo

HEALTH EXAMINATIONS AND THE RIGHT TO HEALTH OF IMMIGRANTS RECENTLY ARRIVED

Instructions:

Your answers will be scanned in a machine. For that reason we ask you:

- Use a blue or black pen, NOT a pencil
- Mark your answers with an X, in this way: ☒ but not like in this: ☒
- If you want to change an answer, fill the box that went wrong: ■ and make a new X in the right one.: ☒
- Write numbers clearly when requested. Write only one number in each box, like this:

1	2	3	4

- Write clear texts in the indicated boxes when explanations are requested or when you want to provide additional information.
- Please, do not write out side of the boxed.

HEALTH EXAMINATIONS AND THE RIGHT TO HEALTH OF IMMIGRANTS RECENTLY ARRIVED

Initially some questions about you and your background

1. In what year were you born?

In 19 ☐☐

2. Are you a woman or a man?

☐ Woman ☐ Man

3. In which country were you born?

☐ Somalia ☐ Afghanistan ☐ Iraq
☐ Syria ☐ Eritrea ☐ Thailand

☐ In another country. Which one? ____________

4. What year did you come to Sweden?

Year ☐☐☐☐

5. When did you obtain residence permission in Sweden?

Year ☐☐☐☐

6. What is your civil status?

☐ Married ☐ Living with partner ☐ Singel
☐ Divorced ☐ Widow(er)

7. Do you have children?

☐ Yes, and they live in Sweden
☐ Yes but they live in another country
☐ No, I do not have children

8. What level of education do you have?

☐ None
☐ 1 – 6 years (Primary school)
☐ 7-12 years (Secondary school)
☐ more than 12 years (High school /University)
☐ Other kind of education, what? ____________

9. What is your religion?

- ☐ I am not a religious person
- ☐ I am a Muslim
- ☐ I am a Christian
- ☐ I am a Buddhist
- ☐ I have another religion. Which?

10. For what reason did you come to Sweden?

- ☐ To apply for asylum.
- ☐ To live with my parents/ family who came earlier to appy for asylum here.
- ☐ To live with / marry a Swede
- ☐ To live with / marry a non-Swedish European living in Sweden
- ☐ To work in Sweden
- ☐ For another reason, what?

Questions about the life in your home country

This questions help us to understand how you lived **the last year** in your home country.

11. What did you mainly do during the last year in your home country?

Mark only one alternative.

- ☐ I worked
- ☐ I did domestic work
- ☐ I was unemployed
- ☐ I did something else, what?
- ☐ I studied
- ☐ I was a farmer

12. Did you and your family have enough money ...

Mark the alternative that best applies to you in each line.

	Yes, always	Yes, sometimes	Yes, sometimes
a. To buy food?	☐	☐	☐
b. To pay school fee and school materials?	☐	☐	☐
c. To pay for health care?	☐	☐	☐

13. How often did you go to consult a doctor (or a nurse), during the last year in your home country?

- ☐ Never
- ☐ In one ocassion
- ☐ More than one ocassion

14. Did you or your family suffered from violence or threats in your home country?

☐ Yes ☐ No ☐ I do not know

15. Did you come to Sweden with your family?

- ☐ Yes, I came here with my family.
- ☐ Yes, I came here with some members of my family.
- ☐ No, I came here alone.

Questions about your first period in Sweden

16. How did you mainly live <u>during the first six months</u> in Sweden?

Mark only one alternative.

- ☐ With other asylum seekers (in a special place for refugees and asylum seekers)
- ☐ With other unaccomapnied children (in an institutions or special place for these children)
- ☐ With family or relatives (at their place)
- ☐ With friends från my country
- ☐ With other persons, who: ____________

17. <u>During your first six months</u> in Sweden...

Mark the alternative that best applies to you in each line.

	Yes, always	Yes, sometimes	No, never
a. Did you have someone who could help with, e.g. contacts with authorities, addresses/find places, translate texts or explain instructions?	☐	☐	☐
b. Did you have someone to talk to about your feelings or personal problems?	☐	☐	☐
c. Did you feel alone or isolated?	☐	☐	☐

18. a. How was your health <u>during the first three months</u> in Sweden?

- ☐ Very well
- ☐ Well
- ☐ Not well, nor bad
- ☐ Bad
- ☐ Very bad

b. Did you need some kind of medical care during the first three months in Sweden?

- ☐ Yes
- ☐ No → **Move to question 19**
- ☐ I do not remember → **Move to question 19**

c. If you answered <u>Yes</u>, what kind of medical care did you need?

- ☐ Due to pregnancy
- ☐ Due to flu
- ☐ Due to chronic desease (e.g. Diabetes)
- ☐ Due to other illness or injury
- ☐ Due to mental desease (e.g. anxiety, depression)
- ☐ Due to an infection (e.g. tuberculosis (TB), HIV/AIDS, Hepatitis)
- ☐ For another reason,which? ____________
- ☐ I do not know

d. Did you get some treatment or care?

- ☐ Yes
- ☐ No
- ☐ I do not know

About health and health care

19. Your ideas about health

Mark the alternative in each line that best applies to you.

	Agree	Disagree	I don't know
a. I can feel bad even if I do not have any disease	☐	☐	☐
b. A person can be sick but feel OK.	☐	☐	☐
c. I can do nothing to influence my own health.	☐	☐	☐
d. God decides about my sickness or health.	☐	☐	☐
e. What I do and how I live have an impact on my health	☐	☐	☐
f. To see a doctor and getting medicines are most important when I am sick.	☐	☐	☐
g. I prefer other alternatives than doctors and medicines when I am sick	☐	☐	☐
h. Illness can be a consequence of sin, magic or evil spirits.	☐	☐	☐

20. Your perceptions about the health care

Mark the alternative in each line that best applies to you.

	Yes	No	I don't know
a. I had better health care services in my country than in Sweden	☐	☐	☐
b. I cannot get here the medicines that doctors would get me in my country.	☐	☐	☐
c. I thrust more to physicians and nurses in my country than I do here.	☐	☐	☐
d. I visit a doctor only if I am seriously sick.	☐	☐	☐
e. For me, physicians should to be of the same sex as myself	☐	☐	☐
f. I avoid talking about sexuality and intimate things, even if the doctor asks me about it.	☐	☐	☐
g. I know where to seek care if I get sick.	☐	☐	☐
h. Physicians in Sweden do not understand my health problems.	☐	☐	☐

21. What source of information is most important for you in relation to food and habits considered good for health?

- ☐ Health care services in Sweden (e.g. doctor, nurse, dietician)
- ☐ Health care in your home country
- ☐ Schools and other institutions for education in Sweden (e.g. SFI)
- ☐ Schools in your home country
- ☐ Media (e.g. TV, internet, books, brochures, posters)
- ☐ Providers of free time activities (e.g. organizations, associations)
- ☐ Relatives and friends
- ☐ Other, specify what: ____________

Questions about health examinations

These questions are about a health examination that new arrivals are offered for free when they come to Sweden, whether healthy or sick. Usually one is invited to the health examination shortly after arrival in Sweden. At the health examination the doctor or nurse often ask about vaccinations, previous illnesses and doing medical tests.

22. Have you ever before heard of health examinations for new arrivals?

- ☐ Yes
- ☐ No → **Move to question 27**

23. From whom did you receive information about the health examination?

You may mark several alternatives.

- ☐ Swedish Migration Board
- ☐ Health care
- ☐ Family, relatives or friends
- ☐ I found the information myself about the health examination.
- ☐ I have not found any information about the health examination. .
- ☐ Others, who?

24. Did you get a letter of invitation to health examination?

- ☐ Yes
- ☐ No → **Move to question 27**

25. a. In what language was the invitation written?

You may mark several alternatives..

- ☐ Swedish
- ☐ English
- ☐ Other language, what?
- ☐ Do not know

b. Did you understand the content?

- ☐ Yes
- ☐ Partly
- ☐ No
- ☐ No, but someone helped me to translate
- ☐ I do not remember

26. What information did you get in the invitation to the health examination?

Mark the alternative that best applies to you in each line.

	Yes	No	Do not remember
a. The purpose of the health examination	☐	☐	☐
b. How the health examination is carried out	☐	☐	☐
c. That the health examination is optional	☐	☐	☐
d. How I could find the way to the health center	☐	☐	☐
e. That the result from the health examination will not be communicated with the Swedish Migration Board	☐	☐	☐

27. In what way would you prefer to receive the invitation to the health examination?

- ☐ Orally
- ☐ Written on paper (letter, brochure etc.)
- ☐ Written on the Internet.
- ☐ By a telephone call
- ☐ SMS sent to my mobile phone
- ☐ On radio
- ☐ In another way, what way? ______

28. What do you think the <u>purpose</u> of the health examination is?

Mark the alternative that best applies to you in each line.

	Yes	No	Do not know
a. To check whether I was healthy or sick	☐	☐	☐
b. To offer care or treatment if I was sick.	☐	☐	☐
c. To identify communicable diseases (e.g. TB, HIV, AIDS, hepatitis)	☐	☐	☐
d. To protect the society from communicable diseases that I may have.	☐	☐	☐
e. To vaccinate and protect me from diseases that might be present in the society	☐	☐	☐
f. To identify if I was fit to start to work	☐	☐	☐
g. To give me information about health issuesr	☐	☐	☐
h. To prevent diseases.	☐	☐	☐

i. Other, What? ______

29. Did you undergo the health examination?

- ☐ Yes
- ☐ No → **Move on to question 42**

30. When did you do the health examination?

- ☐ Less than 1 year ago
- ☐ Between 1 and 2 year ago
- ☐ More than 2 years ago

31. Where did you do the health examination?

- ☐ Stockholm
- ☐ Skåne
- ☐ Östergötland
- ☐ Norrbotten
- ☐ Other place, Where? ____________

32. Why did you do the health examination?

Mark the alternative that best applies to you in each line.

	Yes	No	I do not know
a. It was free of cost.	☐	☐	☐
b. I thought it was compulsory.	☐	☐	☐
c. I wanted to stay in Sweden	☐	☐	☐
d. I was pregnant	☐	☐	☐
e. I was sick and needed health care.	☐	☐	☐
f. I wanted to know if I had any disease.	☐	☐	☐
g. I wanted to know if I had any communicable disease.	☐	☐	☐
h. I needed to talk with someone regarding my health	☐	☐	☐
i. Other persons recommended the health examination	☐	☐	☐

j. Other reason? ____________

33. What expectations did you have regarding undergoing the health examination?

Mark the alternative that best applies to you in each line.

	I agree	I disagree	I do not know
a. It would increase my chance to stay in Sweden	☐	☐	☐
b. To get a diagnos.	☐	☐	☐
c. To get treatment and medicine	☐	☐	☐
d. I had no particular expectations	☐	☐	☐

e. If you had other expectations. What? ____________

34. Who did the health examination?

- ☐ A doctor
- ☐ A nurse
- ☐ Both a doctor and a nurse
- ☐ Do not know
- ☐ Do not remember

About the communication with the doctor or the nurse.

35. When you talked to the doctor o the nurse at the health examination...

Mark the alternative that best applies to you in each line.

	Yes	Partly	No	Do not remember
a. Were you informed on what tests that were done and why?	☐	☐	☐	☐
b. Did you understand the doctor or the nurse?	☐	☐	☐	☐
c. Do you think the doctor or the nurse understood what you were saying?	☐	☐	☐	☐
d. Were your questions answered?	☐	☐	☐	☐
e. Were you satisfied with the answers?	☐	☐	☐	☐
f. Did you get a chance to express your health concerns in a satisfactory way?	☐	☐	☐	☐
g. Did you get any advice regarding your health concerns?	☐	☐	☐	☐
h. Did you get any treatment or medicin?	☐	☐	☐	☐
i. Was there anything you had wanted to talk about during the health examination but did not get a chance to?	☐	☐	☐	☐

j. If **yes** on the last question, what? ____________________

About the content of the health examination

36. During the health examination...

Mark the alternative that best applies to you in each line.

	Yes	No	Do not remember	Do not know
a. Did the doctor or the nurse do a physical examination?	☐	☐	☐	☐
b. Was any medical tests done?	☐	☐	☐	☐
c. Did you get information on what tests that were done?	☐	☐	☐	☐
d. Did you get information on why the tests were done?	☐	☐	☐	☐
e. Did you get the results?	☐	☐	☐	☐

Interpreters

An interpreter is a person who helps with translations when the patient does not speak the same language as the doctor or the nurse.

37. **a. Did you have an interpreter at your health examination?**

- ☐ Yes
- ☐ No ⟶ **Move on to question 38**

b. Were the interpreter present in the clinic or on the telephone?

- ☐ In the clinic
- ☐ On the telephone

c. Did you know the interpreter?

- ☐ Yes, the interpreter was a relative
- ☐ Yes, the interpreter was a friend
- ☐ No, I did not know the interpreter

d. What would you prefer?

- ☐ An interpreter that I know
- ☐ An interpreter that I do <u>not</u> know.
- ☐ Either, it does not matter

e. Was the interpreter of the same sex as you?

- ☐ Yes
- ☐ No
- ☐ I do not know

f. Do you think the interpreter ought to be of the same sex as you?

- ☐ Yes
- ☐ No
- ☐ Either, it does not matter

g. Did you trust the interpreter to translate what you said in a correct way?

- ☐ Yes
- ☐ No

h. How did it work to communicate using an interpreter?

- ☐ It worked well
- ☐ Not well, nor bad
- ☐ Bad
- ☐ Do not know

i. What do you think is the best?

- ☐ To have the interpreter in the room at the health examination
- ☐ To have the interpreter via the telephone

About the right to health

38. Did the doctor or the nurse explain to you any of the following?

Mark the alternative that best applies to you in each line.

	Yes	No	Do not remember
a. That only asylum seekers have the right to the health examination.	☐	☐	☐
b. That it is optional to undergo the health examination.	☐	☐	☐
c. That adult asylum seekers have limited access to health care in Sweden.	☐	☐	☐
d. Those asylum seekers under the age of 18 have the same right to health care as all children in Sweden.	☐	☐	☐
e. What service you may get from the health care.	☐	☐	☐
f. Where you can get health care service if you get sick.	☐	☐	☐
g. Where you may get help if you are feeling very sad, stressed or not being able to sleep.	☐	☐	☐
h. How you protect yourself (or your partner) from an unwanted pregnancy.	☐	☐	☐
i. How you protect yourself from sexually transmitted infections (e.g. HIV/AIDS, Chlamydia och gonorrhea)	☐	☐	☐
j. How to act to reduce the risk to get TB.	☐	☐	☐

39. Your reflection from having done the health examination...

Mark the alternative that best applies to you in each line.

	Yes	Partly	No	Do not remember
a. Did the health examination make you feel better?	☐	☐	☐	☐
b. Did you get information that make you feel better and prevent disease?	☐	☐	☐	☐
c. Did you trust the person who carried out the health examination?	☐	☐	☐	☐
d. Did the health examination correspond to your expectations?	☐	☐	☐	☐
e. Are you over all satisfied with the health examination?	☐	☐	☐	☐
f. Do you think that the doctor or nurse treated you in a respectful manner?	☐	☐	☐	☐
g. Did you feel insulted or derogated in connection to the health examination?	☐	☐	☐	☐

h. If **yes** on the last question, in what way;

40. **If you were treated well and satisfied with the health examination ⟶ Move to 41**

If you were <u>not</u> treated in a good or respectful way at the health examination, what do you think was the reason to this?

Mark the alternative that best applies to you in each line.

	Yes	No	Do not know/ no opinion
a. My language problems / limitations	☐	☐	☐
b. My nationality or ethnic identity	☐	☐	☐
c. My sex	☐	☐	☐
d. My age	☐	☐	☐
e. For being disable or handicapped	☐	☐	☐
f. My religion	☐	☐	☐
g. My colour of skin	☐	☐	☐
h. My sexual orientation	☐	☐	☐

h. Other reason;

41. After the health examination, I feel...

☐ More positive towards the health examination than before.
☐ More negative towards the health examination than before.
☐ Whether more positive or negative

If you did the health examination, go directly to question 44

To you who did NOT do the health examination

42. Why did you not do the health examination?

Mark the alternative that best applies to you in each line.

	Agree	Do not agree	Do not know
a. I was not a asylum seeker	☐	☐	☐
b. I had never heard about the health examination	☐	☐	☐
c. I have not received a letter or invitation	☐	☐	☐
d. I received an invitation but did not want to go	☐	☐	☐
e. I did not understand what the health examination was	☐	☐	☐
f. I thought the health examination may have negative effect on my asylum application	☐	☐	☐
g. I thought I would not get the medicines I needed	☐	☐	☐
h. I do not like to talk about my health problems och difficulties	☐	☐	☐
i. I felt healthy	☐	☐	☐
j. I am afraid of syringes and injections	☐	☐	☐
k. I have bad experiences from contacts with the Swedish Migration Board	☐	☐	☐
l. I have bad experiences from contacts with doctors and nurses in Sweden	☐	☐	☐
m. I was afraid that I had a dangerous disease	☐	☐	☐
n. I did not want to know that I had HIV/AIDS	☐	☐	☐
o. I was afraid for what others might say if I had HIV/AIDS	☐	☐	☐
p. I did not want to know if I had tuberculosis (TB)	☐	☐	☐
q. I was afraid for what others might say if I had tuberculosis (TB)	☐	☐	☐
r. I have heard negative comments on the health examination	☐	☐	☐
s. I was afraid for having to leave Sweden if I was seriously sick	☐	☐	☐
t. Because others I know had not done the health examination	☐	☐	☐

u. Other reason;

43. Do you think of any negative effect by not having done the health examination?

☐ Yes. If so, what?
☐ No
☐ Do not know

Your advices to others in relation to the health examination

44. Would you recommend other persons to do the health examination?

- ☐ Yes
- ☐ No
- ☐ No opinion

Questions regarding your present health status

45. How do you perceive your general health condition during the last three months in Sweden?

- ☐ Very good
- ☐ Good
- ☐ Not good, nor bad
- ☐ Bad
- ☐ Very bad

46. How do you perceive your general health condition in comparison with others in your own age?

- ☐ Better
- ☐ Worse
- ☐ About the same

47. Have you during the last three months been in need for a doctor or seeking health care, but refrained from doing so?

- ☐ Yes
- ☐ No → **Go to question 49**

48. Why did you not go to see a doctor?

- ☐ My problems disappeared
- ☐ Too long waiting time
- ☐ Difficult to get in contact by the telephone
- ☐ Negative experiences from before
- ☐ I did not know where to go or who to ask
- ☐ For economic reasons
- ☐ I did not have the time to go
- ☐ Because of language difficulties
- ☐ Other reason, what? ____________________

About answering the questionnaire

S
ENGELSKA

L

ID

49. How did you answer the questionnaire?

- ☐ I have read by myself and answered the questions in written.
- ☐ Someone read the questions for me and I have then answered the questions in written.
- ☐ Someone read the questions for me and also written the answers according to what I told.

50. Where did you answer the questionnaire?

- ☐ In Stockholm county
- ☐ In Norrbottens county
- ☐ In Östergötlands county
- ☐ In Skåne county

51. Do you have any comments to add?

Thank you very much for your cooperation!
